# Wu-Mei-wan protects pancreatic β cells by inhibiting NLRP3 Inflammasome activation in diabetic mice

**DOI:** 10.1186/s12906-019-2443-6

**Published:** 2019-01-31

**Authors:** Xueping Yang, Fuer Lu, Lingli Li, Jingbin Li, Jinlong Luo, Siyi Zhang, Xinqiao Liu, Guang Chen

**Affiliations:** 1 Institute of Integrative Traditional Chinese and Western Medicine, Tongji Hospital, Tongji Medical College, Huazhong University of Science and Technology, Wuhan, 430030 China; 2 Department of Traditional Chinese Medicine, Puai Hospital, Tongji Medical College, Huazhong University of Science and Technology, Wuhan, 430033 China; 3 Department of Integrative Traditional Chinese and Western Medicine, Tongji Hospital, Tongji Medical College, Huazhong University of Science and Technology, Wuhan, 430030 China; 4 Department of Emergency, Tongji Hospital, Tongji Medical College, Huazhong University of Science and Technology, Wuhan, 430030 China; 5 College of Acupuncture and Moxibustion, Hubei University of Chinese Medicine, Wuhan, 430061 China; 6 School of Pharmceutical Science, South-Central University for Nationalities, Wuhan, 430074 China

**Keywords:** Wu-Mei-wan, NLRP3 inflammasome, Type 2 diabetes mellitus, Pancreatic β cells

## Abstract

**Background:**

Wu-Mei-Wan (WMW) is a traditional Chinese herbal formulation that is clinically prescribed to treat diabetes mellitus in China. WMW has been shown to alleviate damage in pancreatic β cells, but the underlying mechanism remains unclear. This study aims to explore how WMW plays a protective role in pancreatic islets.

**Methods:**

Drug testing and mechanism analyses were performed on mice treated with three concentrations of WMW (4800, 9600, and 19,200 mg/kg/bw) for four consecutive weeks. Blood was collected from both db/db and wild-type mice to determine fasting blood glucose (FBG) and serum insulin levels. The expression of proteins related to apoptosis, cysteinyl aspartate-specific proteinase 12 (caspase-12) and B-cell leukemia 2 (Bcl-2), was measured by western blot. Interleukin-1β (IL-1β), interleukin-18 (IL-18), monocyte chemoattractant protein-1α (MCP-1α), and tumor necrosis factor-α (TNF-α) in the pancreas were tested with enzyme-linked immunosorbent assay (ELISA). Immunohistochemistry staining of F4/80 was performed to measure the pancreatic infiltration of macrophages. Western blot and immunofluorescence staining of the NLRP3 inflammasome were used to measure the expression of proteins related to apoptosis and inflammation.

**Results:**

WMW dose-dependently reduced FBG and promoted serum insulin secretion in db/db mice compared to the wild-type controls. WMW protected pancreatic β cells with a pattern of decreasing caspase-12 and increasing Bcl-2 expression. WMW also reversed the upregulated production of IL-1β, IL-18, MCP-1α, and macrophage-specific surface glycoprotein F4/80 in diabetic mice. In addition, the protein expression levels of NLRP3 inflammasome components NLRP3, ASC, and caspase-1 (P20) were higher in db/db mice than in wild-type controls.

**Conclusions:**

WMW inhibits the activation of the NLRP3 inflammasome to protect pancreatic β cells and prevent type 2 diabetes mellitus development.

**Electronic supplementary material:**

The online version of this article (10.1186/s12906-019-2443-6) contains supplementary material, which is available to authorized users.

## Background

Type 2 diabetes mellitus (T2DM) is a fast-growing health burden. According to the latest report in the International Diabetes Federation Atlas, a total of 425 million adults ranging from 20 to 79 years old, including 212 million undiagnosed adults, are suffering from T2DM worldwide. Another 352 million exhibit impaired glucose tolerance, rendering them vulnerable to T2DM development. China became the country with the largest number of adult patients in 2017, with 114.4 million patients [1]. T2DM is a complex disease characterized by insulin resistance and pancreatic β cell dysfunction. Induced by a high glucose state and saturated fatty acids, as well as chronic inflammation, T2DM leads to pancreatic β cell apoptosis and subsequently insufficient insulin secretion [2]. T2DM is defined as a chronic low-grade inflammatory disease that is closely affected by the secretion of a variety of proinflammatory cytokines [3].

Cross-sectional and prospective studies have revealed elevated circulating C-reactive protein (CRP), interleukin-1β (IL-1β), and interleukin-6 (IL-6) with T2DM [4, 5]. IL-1β is an important chronic proinflammatory cytokine during T2DM development [6]. Ahmad et al reported that interleukin-18 (IL-18) is also associated with an increased risk of insulin resistance and T2DM development [7]. For instance, IL-1β expression is increased in patients with T2DM, whereas inhibition of IL-1β protects β cells from apoptosis, enhanced insulin secretion, and improved insulin sensitivity [6, 8]. IL-1β is secreted by a variety of cells, such as monocytes and macrophages [9]. They are primed upon recognizing motifs carrying pathogen-associated molecular patterns (PAMPs) or damage-associated molecular patterns (DAMPs) to synthesize IL-1β precursors [10]. Adapter molecules and pro-caspase-1 are then recruited to form the inflammasome complex [11]. In the complex, DAMPs function as pattern recognition receptors (PRRs). Toll-like receptors (TLRs), RIG-1-like helicases, nucleotide combining oligomerization domain (NOD) and leucine rich repeat-containing receptors (NLRs) are all important PRRs [12]. The best-characterized inflammasome is the NLR Family Pyrin Domain Containing 3 (NLRP3), which contains a nucleotide-binding site domain, leucine rich repeat motif and pyrin domain (PYD) [13]. Activated NLRP3 inflammasome in the form of the cleaved caspase-1 subunit (p20) cleaves precursors for the maturation of IL-1β and IL-18 molecules [14]. Excessive activation of the NLRP3 inflammasome leads to a number of metabolic disorders, including T2DM [15].

It was also demonstrated that the activation of the NLRP3 inflammasome in infiltrating macrophages can mediate beta cell loss in T2DM [16]. High glucose concentrations induce the production of interleukin-1β in pancreatic β cells, leading to impaired insulin secretion, decreased cell proliferation, and apoptosis [17]. Miao et al reported that the pyroptosis of pancreatic β cells requires activation of caspase-1 [18]. Such islet β cell apoptosis can be reversed in NLRP3 and ASC knockout obese mice [19]. Endoplasmic reticulum stress leads to apoptosis of pancreatic β cells [20], and cysteinyl aspartate-specific proteinase 12 (caspase-12) mediates endoplasmic-reticulum-specific apoptosis [21]. During endoplasmic reticulum stress, overexpression of CHOP promotes the release of mitochondrial cytochrome C and the formation apoptotic body by downregulating the expression of the anti-apoptotic protein B-cell leukemia 2 (Bcl-2), leading to apoptosis [22]. The activation of the NLRP3 inflammasome can decrease the expression of Bcl-2 [23]. Therefore, the NLRP3 inflammasome is a potential target for screening drugs to protect pancreatic β cells and treat T2DM.

Many experiences have been accumulated since diabetes was documented as “Xiao Ke” disease in the classic traditional Chinese medicine book Huang Di Nei Jing Su Wen (91–32 B.C.). Archived in another classic Shang Han Za Bing Lun (200–210 A.D.), Wu-Mei-Wan (WMW) is one of the most important formulations to treat diabetes and has been widely used from ancient times to the present [24, 25]. In addition, many clinical studies demonstrated its benefits [26, 27]. The drug formulation consists of ten herbs and is effective in reducing the fasting blood glucose (FBG) levels of T2DM patients in clinical trials [26]. It has been reported that WMW can improve glucose uptake and decrease FBG in peripheral tissue through the AMP-activated protein kinase (AMPK) pathway [28]. WMW can also alleviate alloxan-induced damage in pancreatic β cells [29]. However, the underlying mechanism is still unclear. Considering that WMW reduces IL-1β expression in patients with inflammatory bowel disease [30] and inhibits the activation of the NLRP3 inflammasome in vitro [31], we hypothesize that WMW protects pancreatic β cells by alleviating the inflammation states through the inhibition of the NLRP3 inflammasome activation and thus prevents T2DM development.

## Methods

### Herbal materials and the preparation of WMW

The herbal components of WMW were purchased from Hubei Traditional Chinese Medicine Co., LTD. (China). This formulation consists of ten herbs, including *Fructus Mume* (*Prunus mume* (Siebold) Siebold & Zucc), *Herba Asari* (*Asarum heterotropoides f. mandshuricum* (Maxim.) Kitag.), *Rhizoma Zingiberis* (*Zingiber officinale* Roscoe), *Rhizoma Coptidis* (*Coptis chinensis* Franch.), *Radix Angelicae Sinensis* (*Angelica sinensis* (Oliv.) Diels), *Rhizoma Typhonii* (*Typhonium giganteum* Engl.), *Pericarpium Zanthoxyli* (*Zanthoxylum bungeanum* Maxim), *Ramulus Cinnamomi* (*Cinnamomum cassia* (L.) J. Presl), *Radix Ginseng* (*Panax ginseng* C. A. Mey), and *Cortex Phellodendri* (*Phellodendron chinense* C. K. Schneid.) (Table 1). Professor Keli Chen from the School of Pharmacy, Hubei University of Chinese Medicine, performed all micro- and macroscopic authentications of the crude drugs to ensure that these met the standards of the Pharmacopoeia of the People’s Republic of China (Edition 2015). All voucher specimens are deposited in the Institute of Integrative Traditional Chinese and Western Medicine, Tongji Hospital, Tongji Medical College, HUST (China) with numbers 2,016,031,001, 2,016,031,002, 2,016,031,003, 2,016,031,004, 2,016,031,005, 2,016,031,006, 2,016,031,007, 2,016,031,008, 2,016,031,009 and 2,016,031,010. The WMW decoction was prepared in the Institute of Integrative Traditional Chinese and Western Medicine, Tongji Hospital, Tongji Medical College, HUST (China).

**Table 1:** The Composition of Wu-Mei-Wan(WMW)

Herbal medicine	Chinese Nane	the Used park	Quantity (g)	Occupied Percent	Disposition Number
Fructus Mume	Wumei	Fruit	24	20%	2016031001
Herbal Asari	Xixin	Root	9	7.5%	2016031002
Rhizoma Zingiberis	Ganjiang	Root	15	12.5%	2016031003
Rhizoma coptidis	Huanglian	Root	24	20%	2016031004
Radix Angclicase	Danggui	Root	6	5%	2016031005
Rhizoma Typhonii Gigantei	Fuzi	Root	9	7.5%	2016031006
Pericarpium Zanthoxyli	Shujao	Pericarp	6	5%	2016031007
Ramulus Cinnamomi	Guizhi	Burgeon	9	7.5%	2016031008
Radix Gensing	Renshen	Root	9	7.5%	2016031009
Cortex Phellodender	Huangbai	Cortex	9	7.5%	2016031010

In brief, *Fructus Mume* (76.8 g) was soaked in vinegar overnight. The root of *Rhizoma Typhonii* (28.8 g) was preboiled for 2 h, while the rest of the herbs (*Herba Asari* 28.8 g, *Rhizoma Zingiberis* 48 g, *Rhizoma Coptidis* 76.8 g, *Radix Angelicae Sinensis* 19.2 g, *Pericarpium Zanthoxyli* 19.2 g, *Ramulus Cinnamomi* 28.8 g, *Radix Ginseng* 28.8 g, and *Cortex Phellodendri* 28.2 g) were soaked in water for 1 h. The final herb mixture was boiled for 2 h. Subsequently, another 1 h boil was performed before filtration. The drug was concentrated to 200 ml by rotating the evaporator, and the concentration was 1.92 g/ml. Then, it was diluted to 0.96 g/ml and 0.48 g/ml with sterile water.

### High-performance liquid chromatography (HPLC) fingerprinting of the extracts

The chemical constituents of WMW extracts were identified by HPLC fingerprinting analysis. Coptisine, ferulic acid, berberine hydrochloride, palmatine hydrochloride, cinnamaldehyde and asarinin were used as standard substances. The extracts were dissolved in water at a concentration of 1.92 g/ml (*w*/*v*) and further diluted with methanol-water (50:50) to 0.96 g/ml (w/v). Samples were passed through an Eclipse XDB-C18 column (4.6 mm × 250 mm, 5 μm) at a flow rate of 1.0 ml/min with the mobile phases of methanol (A) and 0.1% phosphoric acid (B). HPLC signals were recorded at 280 nm detection wavelength with gradient eluting detailed in Table 2.

**Table 2:** Mobile Phase Condition of Chormatographic Separation

Times (min)	Methanol (A)	0.1% Phosphoric Acid
0	5	95
10	5	95
110	55	45
113	5	95
120	5	95

### Drug administration and sample collection

The animal studies were approved by the Animal Ethics Committee of Tongji Medical College, Huazhong University of Science and Technology ([2016] IACUC Number: S306). Twenty-four six-week-old diabetes-prone C57BLKS/J- Leprdb/Leprdb (db/db) male mice and twenty-four six-week-old wild-type (WT) male mice were purchased from the Model Animal Research Center of Nanjing University (China) and housed in a specific pathogen free (SPF) room set at 22 °C ± 2 °C, 55% ± 5% humidity, and a 12/12 circadian rhythm. Water and diet were given ad libitum. After two weeks of adaptive feeding, the db/db mice and WT mice were randomly divided into four groups. The groups were as follows: WT + Vehicle (sterile water), WT + LWMW (4800 mg/kg/bw), WT + MWMW (9600 mg/kg/bw), WT + HWMW (19,200 mg/kg/bw), db/db + Vehicle, db/db + LWMW (4800 mg/kg/bw), db/db + MWMW (9600 mg/kg/bw), db/db + HWMW (19,200 mg/kg/bw), and there were six mice in each group. Intragastric administration was performed to mice once a day for 4 consecutive weeks.

The anesthetic drug sodium pentobarbital (100 mg/kg, Gugeshengwu, Hubei, China) and carbon dioxide euthanasia, minimal environmental irritation, and careful and skillful operations were applied to reduce animal suffering during experimental procedures. After drug anesthesia, blood samples were collected from the mouse orbital venous plexus and transferred into a collection tube, which was then centrifuged at 3000 rpm for 20 min at 4 °C. Serum samples were prepared and kept at − 80 °C for insulin detection. Mice were sacrificed by carbon dioxide euthanasia. The pancreas was cut in half after dissection, half of which was snap-frozen in liquid nitrogen and stored at − 80 °C, and the other half was fixed in 4% paraformaldehyde (PFA).

### Determination of FBG and serum insulin levels

FBG was measured weekly, and blood samples were obtained from the tail vein of mice after fasting for 12 h. Mouse FBG was detected with test strips and glucometer (Roche Co., Ltd., USA) according to the manufacturer’s instructions. After the intervention was completed, the insulin levels of serum samples obtained from the mouse orbital venous plexus were measured by using an insulin enzyme-linked immunosorbent assay (ELISA) kit (American Laboratory Products Company, USA).

### Antibodies and chemicals

Antibodies used in this study were against caspase-12, insulin, and F4/80 from Abcam (UK); against Bcl-2 from Cell Signaling Technology (USA); against IL-1β and ASC from Novusbio (USA); against NLRP3 from R&D (USA); against caspase-1 from Santa Cruz (USA); and against β-actin and β-tubulin from Wuhan Gugeshengwu Technology Co., Ltd. (China). SDS-PAGE gel preparation, phenylmethanesulfonyl fluoride (PMSF), cocktail protease inhibitor, eosin, and hematoxylin were purchased from Wuhan Gugeshengwu Technology Co., Ltd. (China). Antifade mounting medium and 4′,6-diamidino-2-phenylindole (DAPI) were purchased from Biossci Technology Co., Ltd. (China). Kits for tissue protein extraction and BCA protein assay reagent was purchased from Bioyear Biological Technology Co., Ltd. (China).

### Enzyme-linked immunosorbent assay (ELISA)

The pancreas was thawed, weighed and homogenized with 0.9% saline. Then, the homogenate was centrifuged (12,000 g, 15 min, 4 °C), and the supernatant was immediately used for the assessment of IL-1β, IL-18, monocyte chemoattractant.

The protein-1α (MCP-1α) and tumor necrosis factor-α (TNF-α) levels were measured with ELISA kits from Neobioscience Biological Technology Co. Ltd. (China) according to the manufacturer’s recommendations.

### Western blot analysis

Total protein from mouse pancreas homogenates was extracted, separated on 10–12% SDS-PAGE gels (100 V, 1.5 h), and transferred to 0.22 μm PVDF membranes (280 A, 1 kDa/min). Membranes were blocked with 5% nonfat milk for 1 h at room temperature and incubated with primary antibodies overnight at 4 °C. Horseradish peroxidase (HRP)-conjugated secondary antibodies were applied to membranes and incubated for 1 h at room temperature, followed by 3 rounds of interval washes with Tris-buffered saline and Tween 20 (TBST, 10 min each). Bands were visualized using Odyssey Infrared Imaging (LI-COR Biosciences, USA) and normalized against β-actin or β-tubulin using Image Pro Plus software (Media Cybernetics, USA).

### Histology and immunofluorescence staining

After formalin fixation and paraffin embedding, the pancreatic tissues were divided into sections (4 μm). The gross morphology of pancreatic tissue was determined by hematoxylin and eosin staining [32]. Immunofluorescence staining [33] was performed with overnight incubation using diluted primary antibodies against insulin (1:100), caspase-12 (1:100), Bcl-2 (1:100), IL-1β (1:400), ASC (1:200), NLRP3 (1:100), and caspase-1 (1:200) in a humidified chamber at 4 °C. Cell nuclei were counterstained with DAPI. The exposure time of different groups was consistent to obtain the images (CellSens standard system, Olympus, Japan) under the same excitation light.

### Immunohistochemistry staining

Pancreatic tissues fixed in 4% PFA were sectioned after being paraffin embedded. After dewaxing with dimethylbenzene, alcohol gradient and antigen retrieval, pancreatic tissues were treated with 0.3% hydrogen peroxide (H_2_O_2_) in phosphate-buffered saline (PBS) to block endogenous peroxidase activity, followed by blocking in 1% bovine serum albumin (BSA) that was dissolved in PBS. Pancreatic tissue cryosections were incubated with F4/80 antibody (1:100) followed by the Dako REAL™ EnVision™ detection system from Dako (Denmark). The images were acquired using the CellSens standard system. The pictures were analyzed with Image Pro Plus to calculate the average optical density.

### Statistical analysis

Results were presented as mean ± standard deviation (SD) and analyzed using the SPSS 19.0 software (SPSS, USA). Some results are also expressed as percentages to control group. One-way analysis of variance (ANOVA) was used to determine the statistical significance when comparing multiple parameters. Least Significant Difference (LSD) test was performed with the assumption of having equal variances; otherwise, Dunnett’s T3 test was applied. Descriptive and variance homogeneity tests were also ascertained. *P* < 0.05(*) was considered statistically significant.

## Results

### HPLC fingerprinting of WMW

**Fig. 1:**
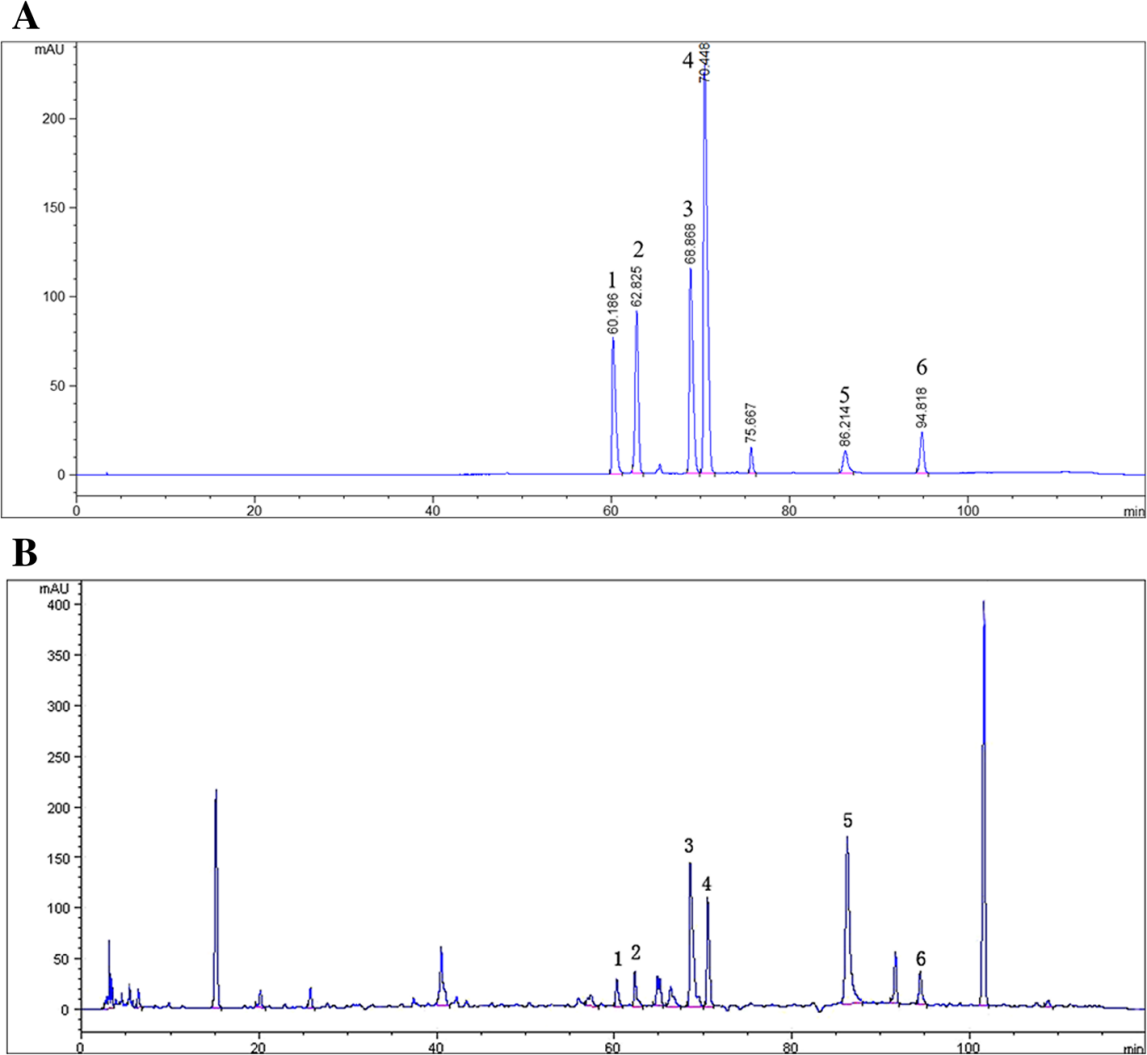
HPLC fingerprint chromatogram of WMW extracts. HPLC fingerprint chromatograms of the extracts of the reference standards (**a**); WMW (**b**). In the chromatograms, (1) coptisine (PubChem CID:72321); (2) ferulic acid (PubChem CID:445858); (3) berberine hydrochloride (PubChem CID:12456); (4) palmatine hydrochloride (PubChem CID:73442); (5) cinnamaldehyde (PubChem CID:637511); and (6) asarinin (PubChem CID:11869417)

The HPLC fingerprint chromatogram of WMW is shown in Fig. 1. Coptisine, ferulic acid, berberine hydrochloride, palmatine hydrochloride, cinnamaldehyde and asarinin were well identified in WMW extracts by comparing both retention times and UV spectra.

### Effect of WMW on FBG and serum insulin levels in db/db mice

**Fig. 2:**
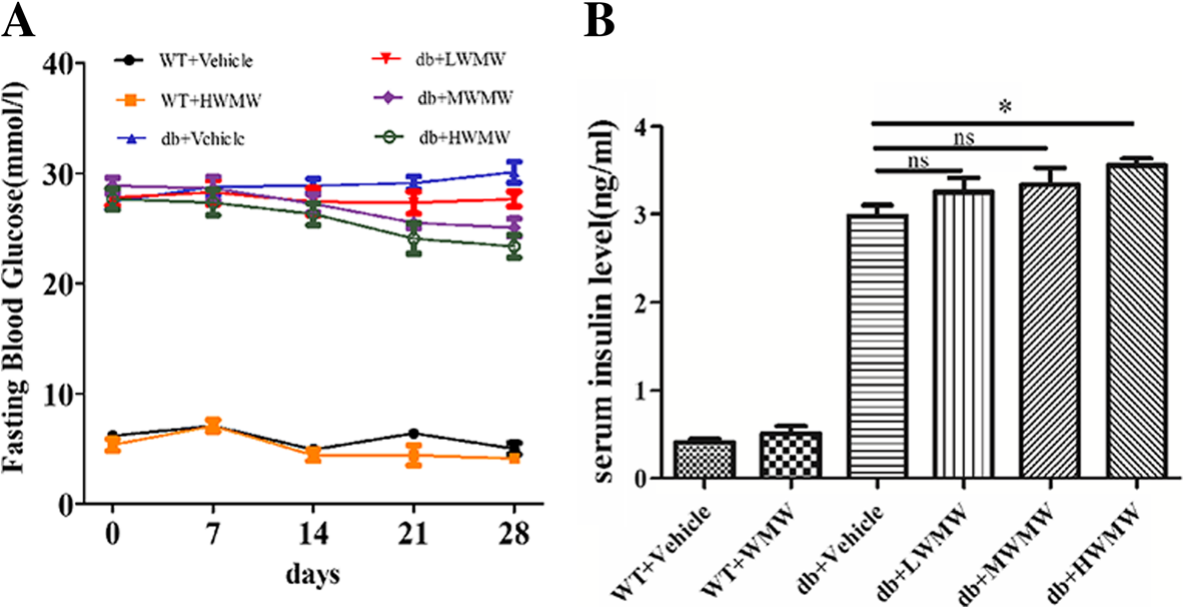
Effect of WMW on FBG and serum insulin levels in db/db mice. Representative graphs were (**a**) FBG (*n* = 6) and (**b**) serum insulin (n = 6). LWMW, MWMW, and HWMW shows mice treated with 4800, 9600, and 19,200 mg/kg/bw WMW, respectively. WT + HWMW shows wild-type mice treated with 19,200 mg/kg/bw WMW, *P* < 0.05(*); no significance (ns)

Blood samples were collected from mice that were treated for four consecutive weeks with three different doses (4800, 9600, and 19,200 mg/kg/bw of WMW). During the course of the intervention, the weekly blood glucose tests showed that the different doses of WMW had no effect on the blood glucose of WT mice. The data of the four groups of WT mice showed no significant differences; only the high dose of WMW in WT mice was selected as the representative data to depict the effect of WMW on WT mice to ensure clarity of Fig. 2a. Although there are 8 groups in our experiment (Additional file 1), six sets of data (WT, WT + HWMW, db/db, db/db + LWMW, db/db + MWMW, db/db + HWMW) are displayed in Fig. 2a. As shown in Fig. 2a, the mice treated with low and medium doses of WMW exhibited lower FBG levels after 21 days compared to the vehicle control group. The mice treated with high WMW concentrations showed a decline in FBG levels as early as day 14. Moreover, it was observed that FBG levels were much lower in wild-type than in diabetic mice. When presented with high-dose WMW treatment, the levels of FBG in wild-type mice remained similar and not statistically significant compared to those of pretreated wild-type mice. Serum insulin levels also remained unaffected in wild-type mice challenged with high-dose WMW. However, serum insulin levels increased in db/db mice under treatment with low and medium WMW doses and was statistically significant in the high dose WMW treatment group (Fig. 2b).

### Effect of WMW on the disordered structure of pancreatic islets in db/db mice

**Fig. 3:**
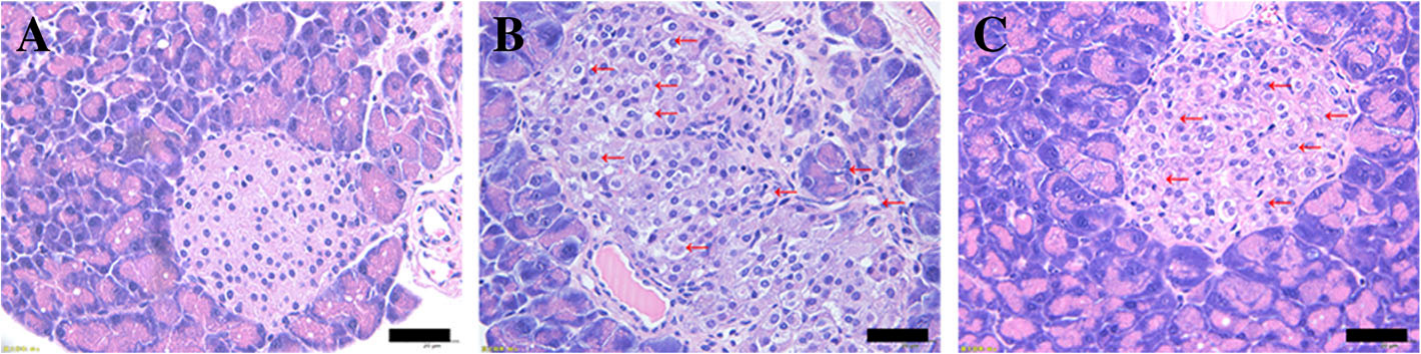
Effect of WMW on the disordered structure of pancreatic islets in db/db mice. Representative photos of H&E staining from the pancreas of (**a**) wild-type; (**b**) db/db; and (**c**) db/db mice treated with HWMW. Scales were 25 μm (*n* = 6)

From the hematoxylin-eosin (HE) staining of pancreas tissue, we demonstrated that vacuolar degeneration with the vague cell boundary was found in the islets of db/db mice (Fig. 3b), defining a hyperplasia and hypertrophy portrait compared to wild-type mice (Fig. 3a). Our results clearly demonstrated that WMW treatment partially rescued the disorganized structure (Fig. 3c).

### Effect of WMW on the expression of proteins related to apoptosis in the mouse pancreas

**Fig. 4:**
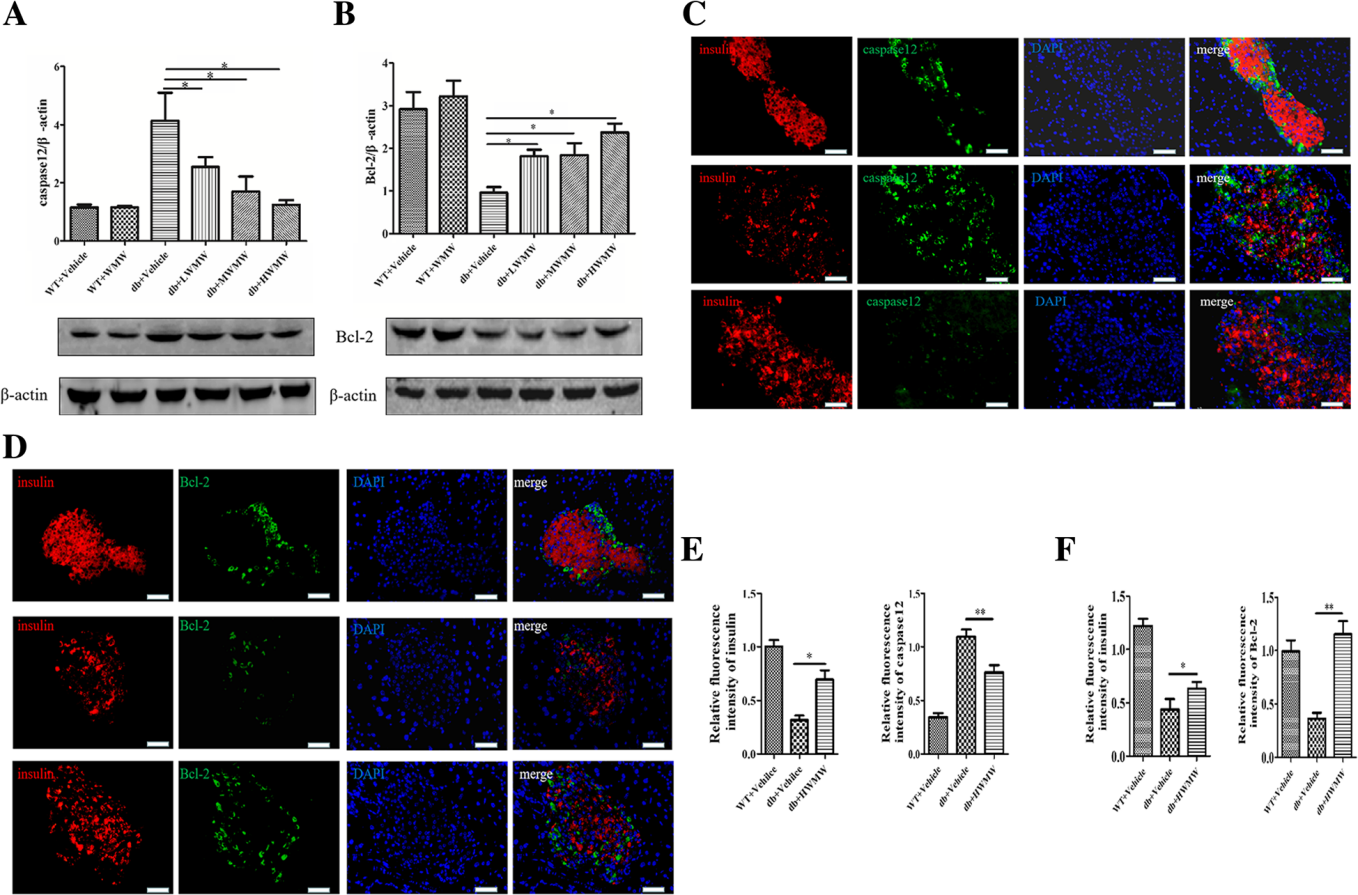
Effect of WMW on the expression of proteins related to apoptosis in the mouse pancreas. Western blot analyses were (**a**) caspase-12 and (**b**) Bcl-2. Band densities were converted to a bar graph. Immunofluorescence staining for insulin (red) and caspase-12 (green) or Bcl-2 (green) in pancreatic islets from wild-type, db/db, and db/db mice treated with HWMW are illustrated in (**c**) or (**d**). Nuclei were counterstained with DAPI (blue). Signals for caspase-12 and Bcl-2 were calculated in the bar graph (**e**, **f**). Scale: 25 μm. *P* < 0.05 (*), *P* < 0.01 (**) (*n* = 6)

To further substantiate our findings that WMW can protect pancreatic β cells, we determined the expression patterns of apoptosis-associated genes in the islets of mice treated with WMW. Diabetic mice synthesized more caspase-12 and less Bcl-2 protein than wild-type controls. This was more likely to lead to higher cell apoptosis. Upon WMW treatment, the expression of caspase-12 was decreased and Bcl-2 was increased. The relationship was found to be WMW dose-dependent. Not surprisingly, caspase-12 and Bcl-2 levels in islets from wild-type mice remained unaffected upon WMW administration (Fig. 4a and b). Our results were further confirmed using immunofluorescence staining, as shown in Fig. 4c-4f. Diabetic mice expressed increased caspase-12 and decreased Bcl-2 and insulin proteins compared with wild-type mice. Treating the mice with WMW significantly reversed the staining outcomes of caspase-12, Bcl-2 and insulin proteins.

### Effect of WMW on the expression of IL-1β and other proinflammatory cytokines in the mouse pancreas

**Fig. 5:**
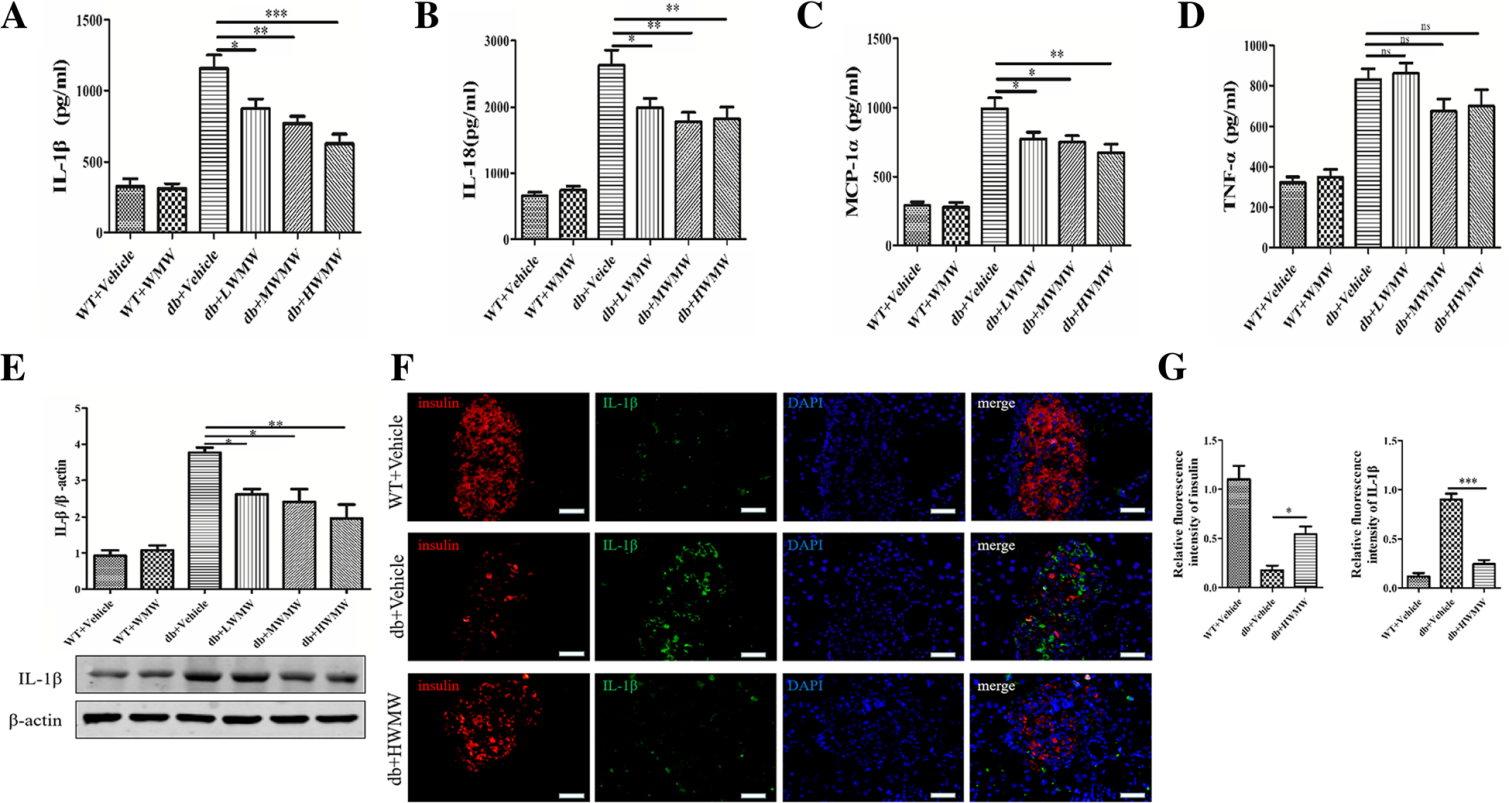
Effect of WMW on the expression of IL-1β and other proinflammatory cytokines in the mouse pancreas. Homogenates of mouse pancreas were tested by ELISA for (**a**) IL-1 β; (**b**) IL-18; (**c**) MCP-1 α; and (**d**) TNF-α. Western blot analysis for IL-1β was performed (**e**). Band densities were converted to a bar graph. Immunofluorescence staining for insulin (red) and IL-1β (green) in pancreatic islets from wild-type, db/db, and db/db mice treated with HWMW are illustrated in (**f**). Nuclei were counterstained with DAPI (blue). Signals for IL-1β were calculated in bar graph (**g**). Scale: 25 μm. *P* < 0.05 (*), *P* < 0.01 (**), *P* < 0.001 (***) (*n* = 6)

Proinflammatory cytokines play an important role in the pathogenesis of T2DM. We performed ELISA on mouse pancreas homogenates and revealed that the pancreas from db/db mice expressed much more IL-1β, as well as IL-18 and MCP-1α, than the wild-type control group. Treating with WMW significantly reduced the production of the aforementioned cytokines in db/db mice. Consequently, no significant differences in the production and secretion of IL-1β, IL-18 and MCP-1α were observed in the wild-type mice treated with WMW (Fig. 5a, b, and c). The high expression level of TNF-α from the pancreas in db/db mice, however, seemed insulated from WMW treatment. Although a slight decrease was observed, it was not statistically significant for the medium to high dose of WMW treatment group (Fig. 5d).

An SDS-PAGE was performed to test the specificity of ELISA on mouse pancreas homogenates for IL-1β. As shown in Fig. 5e, the levels of IL-1β in the pancreas were higher for db/db mice than for wild-type mice. Diabetic mice treated with WMW showed reduced IL-1β production in a WMW dose-dependent manner. Consistent with western blot results, immunofluorescence staining of IL-1β showed increased IL-1β and decreased insulin expression in the islets of db/db mice (Fig. 5f middle) compared to wild-type mice (Fig. 5f top), which was reversed with WMW treatment (Fig. Fig. 5 f bottom). Signals for IL-1β for different conditions were calculated and are shown in Fig. 5g.

### Effect of WMW on macrophage infiltration in the mouse pancreas

**Fig. 6:**
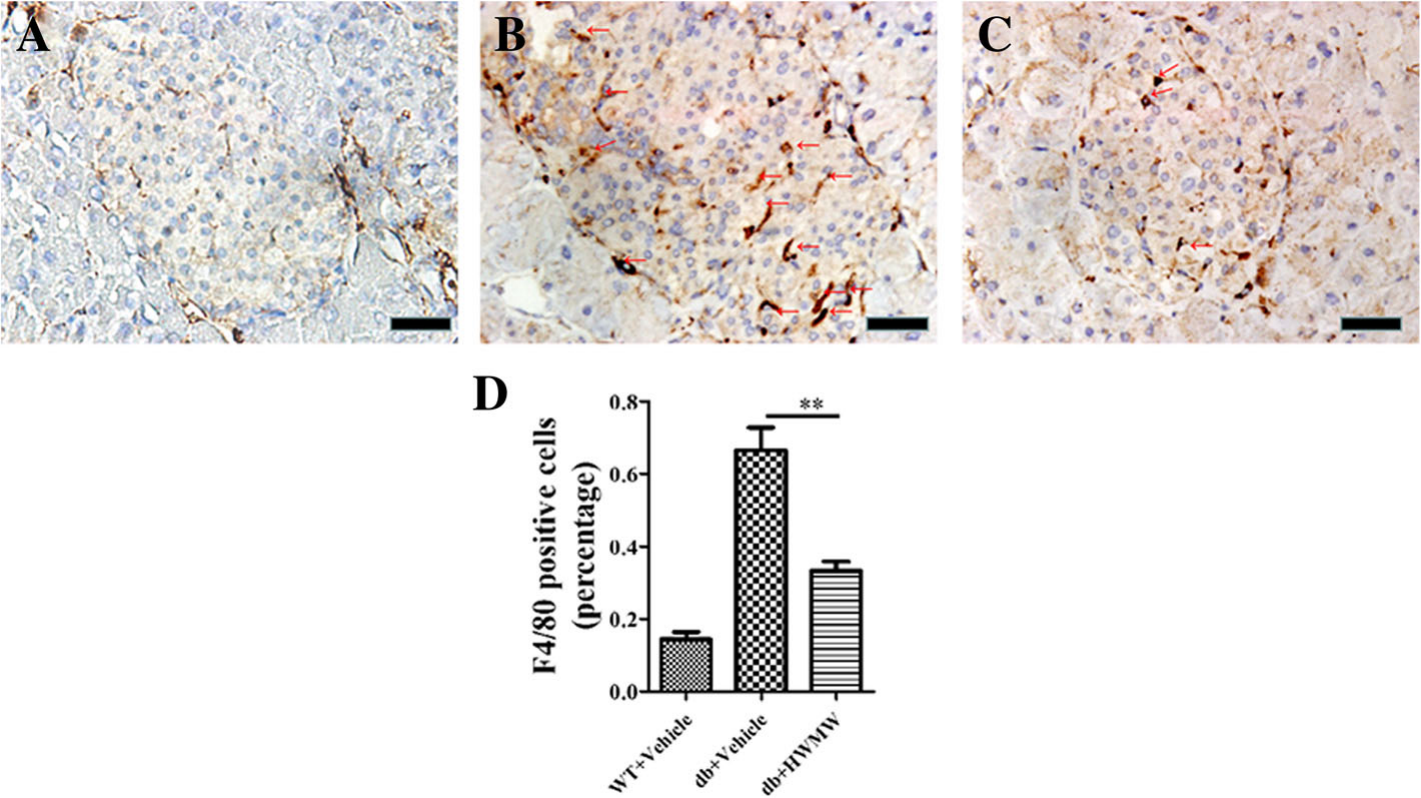
Effect of WMW on macrophage infiltration in the mouse pancreas. Immunohistochemistry staining for F4/80 in mouse islets from the pancreas of (**a**) wild-type; (**b**) db/db; (**c**) db/db mice treated with HWMW and (**d**) the bar graph for percentages of F4/80 positive cells (red arrows). Scale: 25 μm. *P* < 0.01 (**) (*n* = 6)

Both IL-1β and IL-18 are important cytokines produced and secreted by M1 (inflammatory) macrophages. Since WMW treatment decreased IL-1β and IL-18 expression in db/db mice, we further evaluated if WMW affected macrophage infiltration into the islets. We performed immunohistochemistry staining on macrophage-specific surface glycoprotein F4/80. It was determined that islets from db/db mice developed much more macrophage positive staining than wild-type control mice, which was attenuated by WMW treatment (Fig. 6).

### Effect of WMW on the activation of the NLRP3 inflammasome in the mouse pancreas

**Fig. 7:**
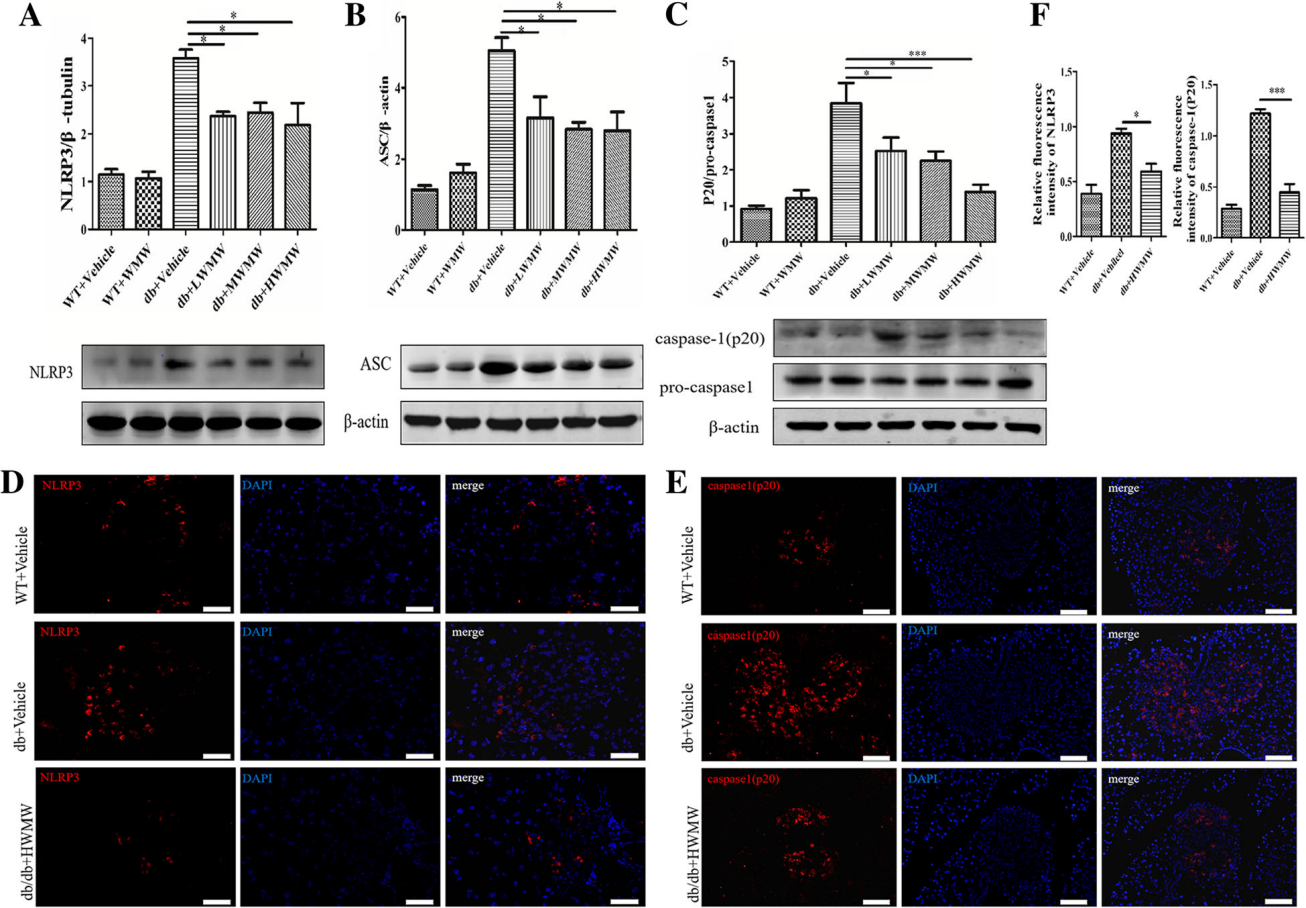
Effect of WMW on the activation of the NLRP3 inflammasome in the mouse pancreas. Western blot analyses for NLRP3 (**a**); ASC (**b**); and caspase-1 (P20) (**c**). Band densities were converted to a bar graph. Immunofluorescence staining for NLRP3 (red) and caspase-1 (P20) (red) in pancreatic islets from wild-type, db/db, and db/db mice treated with HWMW (19,200 mg/kg/bw) are illustrated in (**d**) and (**e**), respectively. Nuclei were counterstained with DAPI (blue). Scales were 25 μm (**d**) and 50 μm (**e**). Signals for NLRP3 and caspase-1 (P20) were calculated in bar graph (**f**). *P* < 0.05 (*), *P* < 0.001 (***) (*n* = 6)

Mature IL-1β is produced via NLRP3 inflammasome-mediated cleavage of its precursor. Considering its significance, we tested whether the activation of the NLRP3 inflammasome was hampered by WMW uptake in db/db mice. The expression levels of NLRP3, the adapter protein ASC, and the functional executor component within the inflammasome, caspase-1 (P20), were increased in db/db mice compared to wild-type controls, suggesting diabetic activation of the NLRP3 inflammasome. As expected, WMW treatment significantly reduced the expression of the NLRP3 inflammasome in diabetic mice (Fig. 7a, b, and c). Consistently, the inflammasome components NLRP3 and IL-1β developed an increased immunofluorescence staining pattern in db/db mice compared to wild-type control groups, which was reduced upon WMW treatment (Fig. 7d-7f).

## Discussion

WMW is a traditional Chinese herbal formulation that shows great promise in the treatment of T2DM. Although its underlying mechanism is unclear, WMW has long been used as a prescription for diabetes in China. Malic acid, citric acid, and succinic acid isolated from *Fructus Mume,* which is the main component of WMW, participate in the Krebs cycle and affect the adenosine monophosphate (AMP) and adenosine-triphosphate (ATP) status [34], the latter of which is considered an agonist to the NLRP3 inflammasome. Among the other components, *Rhizoma Coptidis* has been validated in diabetes treatment [35, 36] by decreasing blood glucose levels. Malonylginsenosides, a natural ginsenoside of *Radix Ginseng*, alleviates hyperglycemia and hyperlipemia of T2DM [37]. *Ramulus Cinnamomi* contains cinnamic aldehyde. Cinnamic aldehyde improves leptin expression [38], which is important because leptin receptor-deficient db/db mice exhibit increased FBG and apoptotic β cells in T2DM [39].

Several components of WMW identified by HPLC have also been shown to have anti-diabetic effects. It has been demonstrated that coptisine not only has a lipid-lowering effect [40] but also protects the rat heart by suppressing myocardial apoptosis and inflammation [41]. Ferulic acid is an antioxidant and anti-inflammatory agent [42–44] and can be used for the treatment of T2DM in diabetic rats [45]. In both animal experiments [46] and clinical trials [47], berberine has shown a good hypoglycemic effect for T2DM. Cinnamaldehyde can modulate proinflammatory cytokines and oxidative stress [48], decrease lipid accumulation [49] and treat diabetes [50]. Asarinin is can inhibit the expression of the apoptosis-related proteins caspase-3 and BAX [51]. In addition, it can influence the Toll-like pathway, which plays an important role in diabetes [52].

The db/db mice in this study were spontaneous diabetes model mice with increasing plasma insulin and blood glucose from 10 to 14 days after birth. Under the C57BLKS genetic background, a number of characteristics can be observed, including the loss of beta cells in the islets that produce insulin and uncontrolled increases in blood glucose [53]. Our results showed that WMW selectively decreased FBG levels in db/db mice but had no impact on normal mice. This is in concordance with the classic theory of traditional Chinese medicine and supports the practical rationales for clinical WMW prescription. Potentially, WMW could be superior over current hypoglycemic agents to reduce the risk of therapy-driven hypoglycemia. Inflammatory responses are the body’s protective mechanism to eliminate microbial infections, but such activations may not always benefit the organism. Sustained inflammation can adversely lead to chronic disease progression, including atherosclerosis, arthritis, and diabetes. In these scenarios, the delicate balance of cytokine secretion is interrupted. IL-1β and IL-18 are two interleukins from the cytokine lists that are believed to play important roles in the pathogenesis of diabetes [54]. Maedler et al detected apoptosis related to factor-associated suicide (FAS) in islet β cells from patients with T2DM [55]. A later study reported that IL-1β increased FAS expression [56], which activated the caspase-related apoptotic cascade in islet β cells. Sustained high glucose exposure had been reported to interfere with insulin secretion and induce pancreatic β cell apoptosis in an IL-1β-dependent manner [57]. Treating β cells with IL-1β antagonists not only prevents programmed cell death but also potentiates glucose-induced insulin secretion and improves insulin sensitivity [57]. Our current study showed that WMW protected the pancreas in db/db mice with reduced IL-1β and IL-18. This in turn led to specific downregulation of caspase-12 and upregulation of anti-apoptotic Bcl-2 expression.

Mature IL-1β and IL-18 can be processed via inflammasome-mediated proteolytic cleavage of their precursors. NLRP3 is the largest inflammasome subset in cells and can be activated by both pathogenic microorganisms (such as viruses, fungi, and bacteria) and cell metabolic products (extracellular ATP, crystalline uric acid, hyperglycemia, hyperlipidemia, etc.). The activating signals are transduced via activation of NF-**κ**B and its subsequent nuclear translocation [58]. TNF-α has been reported to trigger this process [59]. In terms of expression levels, our results demonstrated that WMW did not alter TNF-α expression in the pancreas of db/db mice. Further investigations are needed to determine whether WMW can functionally activate TNF-α. We postulate that once activated by NF-**κ**B, there are three potential mechanisms for the assembly of the NLRP3 inflammasome. These are through the ATP channel, lysosomal and reactive oxygen species (ROS) pathways. Interestingly, the auxiliary activation agent of inflammasome thioredoxin-interacting protein (TXNIP) involved in ROS is closely related to the occurrence of diabetes [60]. As a strong facilitator, a high concentration of glucose boosts TXNIP expression and activates the NLRP3 inflammasome in pancreatic cells. It remains to be tested whether TXNIP is activated during WMW treatment, but it has been reported that at least three components of WMW (*Radix Ginseng*, *Rhizoma Coptidis,* and *Cortex Phellodendri*) have antioxidant effects [61–63]. Assembled NLRP3 oligomerizes in the presence of activating signals, exposing its PYD for interaction with adaptor protein ASC. Such an interaction in turn recruits and cleaves pro-caspase-1, releasing activated caspase-1 for the maturation of IL-1β and IL-18 [64].

Activation of the NLRP3 inflammasome causes severe liver inflammation and pyroptotic death of hepatocytes [65]. It also induces cell apoptosis in the vascular endothelium [66]. Moreover, NLRP3 activation is closely related to cell death and T2DM. Activation of the NLRP3 inflammasome is important for islet amyloid polypeptide (IAPP)-mediated cell death in the pancreas [67]. It promotes IL-1β secretion and induces the apoptosis of pancreatic β cells [68]. On the other hand, mice lacking the NLRP3 inflammasome exhibit improved glucose tolerance against a high-fat diet [69]. NLRP3 knockout mice have lower FBG levels as well as less IL-1β and IL-18 infiltration in the liver and adipose compared to their wild-type control [70]. Our results showed that WMW inhibited IL-1β expression, possibly due to interrupted NLRP3 activation. Interestingly, we found that WMW decreased protein expression not only in NLRP3 but also in other inflammasome components, such as ASC and caspase-1, in the pancreas of diabetic mice.

## Conclusion

In conclusion, our study worked on the mechanism underlying WMW treatment for T2DM and revealed that WMW attenuated the degree of inflammation through the inhibition of NLRP3 inflammasome activation. This provided protection for pancreatic β cells and eventually impeded T2DM. Our study supports the use of WMW as a clinically prescribed drug for T2DM and serves as the foundation to further investigate its chemical components for the development of new drugs to better manage the disease.

## Additional file

Additional file 1: Effect of WMW on FBG and serum insulin levels in db/db mice. Representative graphs were (A) FBG (*n* = 6); and (B) serum insulin (n = 6). LWMW, MWMW, and HWMW shows mice treated with 4800, 9600, and 19,200 mg/kg/bw WMW, respectively. *P* < 0.05(*); no significance (ns). (PDF 324 kb)

## Abbreviations

AMP: Adenosine monophosphate
AMPK: AMP-activatedproteinkinase
ASC: Apoptosis-associated speck-like protein containing a CARD domain
ATP: Adenosine-triphosphatetriphosphate
Bcl-2: B-cell leukemia 2
caspase1: Cysteinyl aspartate specific proteinase 1
caspase12: Cysteinyl aspartate specific proteinase 12
CRP: C-Reactive protein
DAMPs: Danger associated molecular patterns
DAPI: 4′,6-diamidino-2-phenylindole
db/db: C57BLKS/J-Lepr^db^/Lerp^db^
ELISA: Enzyme-linked immuno sorbent assay
FAS: Factor associated suicide
FBG: Fasting blood glucose
HPLC: High performance liquid chromatography
IAPP: Islet amyloid polypeptide
IL-18: Interleukin-18
IL-1β: Interleukin-1β
IL-6: Interleukin-6
MCP-1α: Monocyte chemoattractant protein-1α
NF-κB: Nuclear factor-κ-gene binding
NLRP3: Nucleotide-binding oligomerization domain, leucine rich repeat and pyrin domain containing 3
NLRs: Leucine rich repeat-containing receptor
NOD: Nucleotide combining oligomerization domain
PAMPs: Pathogen associated molecular patterns
PFA: Paraformaldehyde
PKB: Protein kinase B
PMSF: Phenylmethanesulfonyl fluoride
Pro-IL-1β: precursor of interleukin-1β
PRRs: Pattern recognition receptors
PYD: Pyrin domain
ROS: Reactive oxygen species
SPF: Specific pathogen free
T2DM: Type 2 Diabetes Mellitus
TBST: Tris-Buffered-Saline and Tween 20
TLRs: Toll-like receptors
TNF-α: Tumor necrosis factor-α
TUNEL: Td-mediated dUTP nick end Labeling
TXNIP: Thioredoxin-interacting protein
WMW: Wu-Mei-Wan
WT: wildtype
