# Supplementary figures and images for: Wu-Mei-wan protects pancreatic β cells by inhibiting NLRP3 Inflammasome activation in diabetic mice
Source: BMC Complement Altern Med. 2019 Jan 31;19:35. doi: 10.1186/s12906-019-2443-6 (PMC6357370; doi:10.1186/s12906-019-2443-6)

A

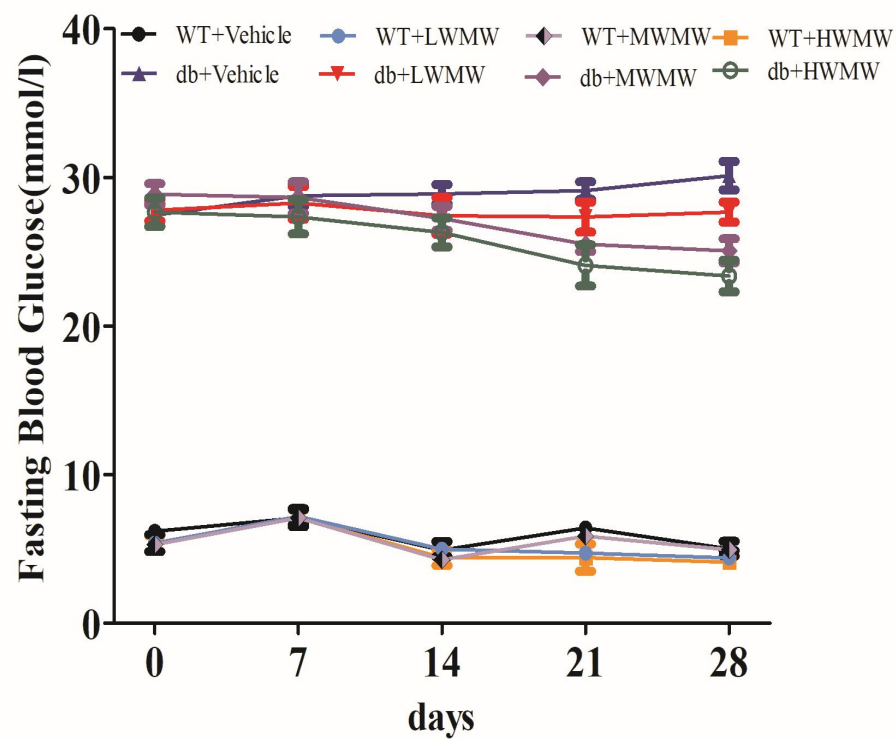

B

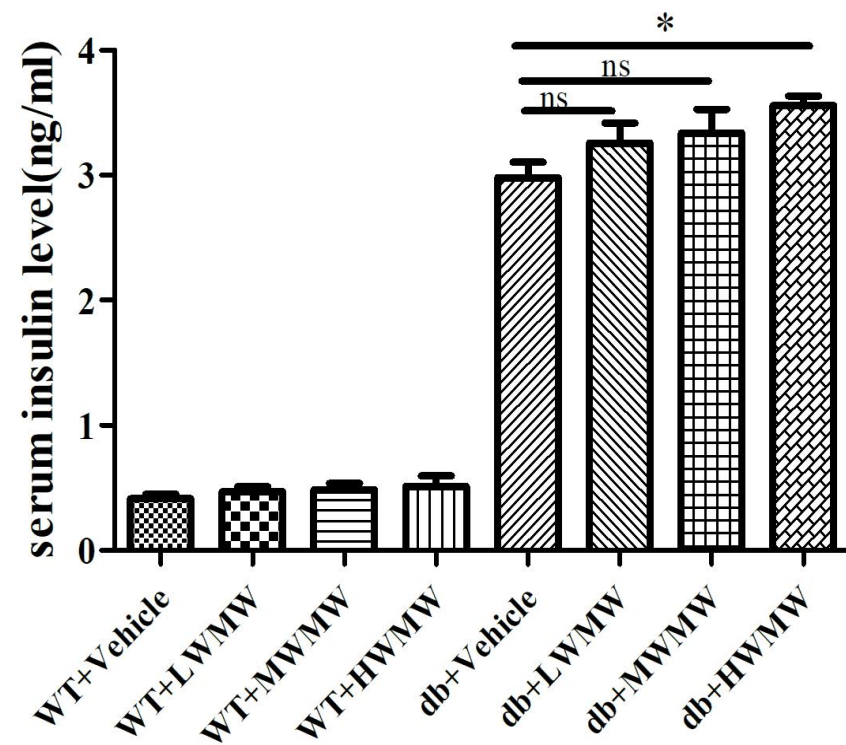

Supplement: Supplementary file 1 — Effect of WMW on FBG and serum insulin levels in db/db mice. Representative graphs were (A) FBG (n = 6); and (B) serum insulin (n = 6). LWMW, MWMW, and HWMW shows mice treated with 4800, 9600, and 19,200 mg/kg/bw WMW, respectively. P < 0.05(*); no significance (ns). (PDF 324 kb) [file 12906_2019_2443_MOESM1_ESM.pdf]
